# Mice Overexpressing Both Non-Mutated Human *SOD1* and Mutated *SOD1^G93A^* Genes: A Competent Experimental Model for Studying Iron Metabolism in Amyotrophic Lateral Sclerosis

**DOI:** 10.3389/fnmol.2015.00082

**Published:** 2016-01-06

**Authors:** Anna Gajowiak, Agnieszka Styś, Rafał R. Starzyński, Aleksandra Bednarz, Małgorzata Lenartowicz, Robert Staroń, Paweł Lipiński

**Affiliations:** ^1^Department of Molecular Biology, Institute of Genetics and Animal Breeding, Polish Academy of SciencesMagdalenka, Poland; ^2^Department of Genetics and Evolution, Institute of Zoology, Jagiellonian UniversityKraków, Poland

**Keywords:** ALS, SOD1, G93A, neurons, iron, skeletal muscle, heme oxygenase 1, oxidative stress

## Abstract

Amyotrophic lateral sclerosis (ALS) is a progressive neurodegenerative disease characterized by degeneration and loss of motor neurons in the spinal cord, brainstem and motor cortex. Up to 10% of ALS cases are inherited (familial, fALS) and associated with mutations, frequently in the superoxide dismutase 1 (*SOD1*) gene. Rodent transgenic models of ALS are often used to elucidate a complex pathogenesis of this disease. Of importance, both ALS patients and animals carrying mutated human *SOD1* gene show symptoms of oxidative stress and iron metabolism misregulation. The aim of our study was to characterize changes in iron metabolism in one of the most commonly used models of ALS – transgenic mice overexpressing human mutated *SOD1^G93A^* gene. We analyzed the expression of iron-related genes in asymptomatic, 2-month-old and symptomatic, 4-month-old *SOD1^G93A^* mice. In parallel, respective age-matched mice overexpressing human non-mutated *SOD1* transgene and control mice were analyzed. We demonstrate that the overexpression of both *SOD1* and *SOD1^G93A^* genes account for a substantial increase in SOD1 protein levels and activity in selected tissues and that not all the changes in iron metabolism genes expression are specific for the overexpression of the mutated form of SOD1.

## Introduction

Amyotrophic lateral sclerosis (ALS) is the most widespread motor neuron disease. It is characterized by a progressive and selective degeneration of neurons in motor cortex and lower motor neurons projecting from the brainstem and spinal cord. In consequence, the disease leads to gradual skeletal muscle weakness, atrophy and paralysis and it is usually fatal within 2–3 years from the onset. The most common cause of death among ALS patients is respiratory failure caused by the degeneration of the nerves and muscles that control breathing (Pratt et al., 2012). Although ALS is mostly a sporadic disease (sALS) of generally unknown etiology, approximately 10% of ALS cases, designed as familial ALS (fALS), are due to genetic factors and have an autosomal dominant pattern of transmission (Rothstein, 2009). Mutations found within the *SOD1* gene encoding an antioxidant enzyme, (SOD1, CuZn-SOD) were the first established genetic cause of ALS (Rosen et al., 1993) and are nowadays estimated to account for up to 20 and 3% of fALS and sALS cases, respectively. In addition to *SOD1* gene mutations, defects in some other genes have also been involved in fALS (Pratt et al., 2012). Importantly, how the various ALS-linked gene products determine similar course of the disease is incompletely understood. The two forms of ALS (fALS and sALS) share similar clinical and pathological features, suggesting common molecular pathogenic mechanisms. Oxidative stress (a condition arising from an imbalance between the production of reactive oxygen species and the efficiency of antioxidant defense systems) has been proposed to be implicated in ALS pathogenesis mostly by interfering with other pathogenic mechanisms, such as motor neuron excitotoxicity, mitochondrial and cytoskeletal dysfunction, protein aggregation, neuroinflammation, and deficit in neutrophic growth factors (Barber et al., 2006). The evidence of the involvement of oxidative stress in ALS pathology originates from studies on ALS patients (Carri et al., 2003) as well as from experiments on rodent models of this disease, which are in vast majority transgenic mice that overexpress mutated human *SOD1* gene (Turner and Talbot, 2008). Although in general these mouse models recapitulate human disease, some critical differences exist between the animal and human pathology. An important one concerns an increased enzymatic activity of the SOD1 in some transgenic mice widely used in ALS research (for example *SOD1^G37R^* and *SOD1^G93A^*), a phenomenon, which does not occur in humans. Of importance, SOD1 is a particular antioxidant enzyme as it converts the superoxide anion radical (O_2_^⋅-^) to another reactive oxygen species (ROS) – hydrogen peroxide (H_2_O_2_) (McCord and Fridovich, 1969). It seems therefore that the final effect of SOD1 overexpression depends on the balance between the beneficial effects of the O_2_^⋅-^ scavenging and the destructive effects of H_2_O_2_ production. In this context, it is not surprising that the toxicity induced by oxidative stress has been reported in both, SOD1 null mice (Reaume et al., 1996) and in mice overexpressing human wild-type *SOD1* gene (Lee et al., 2001).

The commonly accepted paradigm of iron dichotomy in biological systems states that iron is essential for the function of many enzymes and thus it is absolutely fundamental for most biological life forms, but, on the other hand, it is toxic in excess (Pantopoulos et al., 2012). Toxicity of iron is usually explained by its ability to induce oxidative stress through the catalysis of the Fenton reaction that leads to the formation of the hydroxyl radical (⋅OH), a highly destructive oxidant. Both aforementioned ROS interact with iron in the Fenton reaction: O_2_^⋅-^ as a rate-limiting reducing factor for the pre-existing pool of free iron active in the generation of ⋅OH, and H_2_O_2_, as a factor that directly reacts with ferrous iron to yield this radical. Apart from the participation in the Fenton reaction, both H_2_O_2_ and O_2_^⋅-^ have been shown to alter the expression of iron-related genes by various molecular mechanisms (reviewed by Orino et al., 2001).

Misregulation of iron homeostasis in the central nervous system, which results in the pathological iron accumulation and in increased formation of ROS is a frequent phenomenon, largely documented in neurodegenerative diseases (Oshiro et al., 2011; Hadzhieva et al., 2014). The elevated amounts of iron deposits have been also reported in the brain and spinal cord of ALS patients (Kasarskis et al., 1995; Kwan et al., 2012; Ignjatović, 2013) and mice overexpressing mutated human *SOD1* gene (Jeong et al., 2009; Lee et al., 2015). Presumably, the most convincing argument supporting pathological involvement of iron accumulation and iron-mediated oxidative stress in the progression of ALS derives from studies showing beneficial effects of iron chelation therapy in transgenic mice overexpressing human mutated *SOD1* gene (Jeong et al., 2009; Kupershmidt et al., 2009; Wang et al., 2011; Lee et al., 2015). Based on the analysis of the complex interplay between oxidative stress and disturbed iron homeostasis in these models, Hadzhieva et al., 2014 proposed that in ALS, oxidative stress strongly affects cellular iron balance leading to iron overload in motor neurons, and thus creates a vicious circle to exacerbate oxidative injury. However, it remains unclear, up to what extent SOD1 mouse models of ALS reproduce the mechanisms of oxidative stress induction in human pathology. In particular, an intriguing question remains open: how (if at all) the increased SOD1 activity observed in the CNS of transgenic *SOD1^G93A^* and *SOD1^G37R^* mice affects their iron metabolism. Of importance, to our knowledge, only these two mouse models were used so far in *in vivo* studies of iron metabolism dysregulation in ALS. Strictly speaking, it is still unclear, which iron metabolism dysregulation mechanisms can be regarded as a disease-specific (and thus can be used to draw conclusions in humans), and which should be regarded as a secondary, model-associated effects.

Here, to clear up this issue, in addition to mice overexpressing human mutated *SOD1^G93A^* gene we used two types of control age-matched mice – wild-type mice and mice overexpressing human wild-type *SOD1* gene. In those mice we compared the expression pattern of iron-related genes in their brain stems (*Medulla oblongata*), spinal cords, skeletal muscles (represented by *gastrocnemius* and broadest dorsal – *Latissimus dorsi* muscles) and livers. We have also determined iron content and distribution in their *gastrocnemius* muscles.

We demonstrate that the overexpression of both *SOD1* and *SOD1^G93A^* genes accounts for a substantial increase in SOD1 protein levels and activity in selected tissues and that not all the changes in iron metabolism genes expression are specific for the overexpression of the mutated form of SOD1. Furthermore, not all the changes are specific to the disease-affected human tissues. Importantly, among various analyzed genes, only *Hmox1*, encoding HO1, an important oxidative stress responder was found to be induced solely in mice overexpressing human mutated *SOD1* gene and only in tissues known to be affected by ALS. Similarly, iron accumulation in the *gastrocnemius* muscle was also exclusively restricted to mice carrying *SOD1^G93A^* mutation in the symptomatic stage of disease.

## Materials and Methods

### Mice

Following mice provided by The Jackson Laboratory (Bar Harbor, ME, USA) were used in the study: males [strain B6SJL-Tg(SOD1-G93A)1Gur/J] hemizygous for the *SOD1^G93A^* transgene, with transgenic expression of a G93A mutant form of human SOD1 (harboring a single amino acid substitution of glycine to alanine at codon 93) (Gurney et al., 1994), transgenic males [strain B6SJL-Tg(SOD1)2Gur/J] carrying the normal allele of the human *SOD1* gene (called *SOD1)*, and males (strain B6129PF2/J) used as controls for genetically engineered mice from the above mentioned strains. *SOD1^G93A^* mice exhibit a phenotype similar to ALS in humans. All analyses were performed in age-matched 2- and 4-month-old mice, designed as asymptomatic and symptomatic (showing apparent symptoms of paralysis in the case of mice overexpressing human mutated gene), respectively. The age of mice from the two control groups (wild-type and mice overexpressing human wild-type SOD1) was the same as for the ALS model mice. Mice arrived at the age of 4 weeks and were housed at 24–25°C, relative humidity 50–60% with a light–dark cycle of 12 h. Mice received a standard diet (Labo-feed, Kcynia, Poland) and water *ad libitum*. Animals were euthanized by intraperitoneal injection of Vetbutal (Biovet, Puławy, Poland). All procedures were conducted according to the guidelines of the Directive 2010/63/EU of the European Parliament and of the Council of 22 September 2010 on the Protection of Animals Used for Scientific Purposes.

### Measurement of SOD1 Activity

Superoxide dismutase 1 activity in tissue total extracts was measured by gel electrophoresis using the Nitroblue Tetrazolium (NBT)/riboflavin method as described previously (Beauchamp and Fridovich, 1971). Briefly, 15 mg samples of the tissue total extracts were resolved by electrophoresis on 12% polyacrylamide gels under non-denaturing and non-reducing conditions. After electrophoresis, the activity of SOD was visualized by immersion of the gels in staining buffer [50 mM potassium phosphate (pH 7.8), 0.1 mM EDTA, 28 mM TEMED, 3 mM riboflavin, 0.25 mM NBT] for 30 min in the dark at RT. Gels were then exposed to light until the SOD activity bands became visible as bright bands on a dark blue background. The reaction was stopped by rinsing the gels with water. Activities of CuZn-SOD, and Mn-SOD were distinguished by a selective inhibition of the former activity by incubation of gels in a buffer containing 3 mM KCN prior to the staining, as described by Salin and Bridges (1981).

### Hematoxylin/Eosin and Prussian Blue Staining of the *gastrocnemius* Muscle

The *gastrocnemius* muscle was excised from both hind limbs, dissected from fat and connective tissue, immediately fixed in Bouin’s solution for 24 h, and stored in 70% ethanol before further preparation. After dehydration, muscle was embedded in paraffin and cut into 7 μm sections with a microtome (Reichert-Jung, Germany) on the horizontal plane, transversely to the long axis of the muscle. The sections were placed on a slide and stained with haematoxylin and eosin. The morphology of muscle structure was studied by standard light microscopy (Olympus, type CH2) under × 2 and × 40 objectives.

Non-heme iron staining of the *gastrocnemius* muscle samples was analyzed using Accustain Iron Deposition Kit (Sigma–Aldrich). After mounting on glass slides, muscle sections were deparaffinized, incubated with working solution containing Perls’ Prussian blue for 30 min, counterstained with pararoseaniline solution for 2 min and analyzed under standard light microscopy (Olympus, type CH2).

### RNA Preparation and Real-Time Quantitative RT-PCR

Total RNA was extracted using the TRIzol reagent (Invitrogen). 0.5 μg (spinal cord), 1 μg (gastrocnemius) or 2 μg (liver, broadest dorsal muscle) of total RNA was reverse-transcribed using random hexamers and Transcription First Strand cDNA Synthesis Kit (Roche). Real-time quantitative PCR was performed using the Roche Light Cycler 96 system and the FastStart Essential DNA Green Master Kit (Roche) together with the following primers: TfR1, 5′-TCG CTT ATA TTG GGC AGA CC-3′ (forward) and 5′-CCA TGT TTT GAC CAA TGC TG-3′ (reverse); HO1, 5′-TCT TGC CTG GCT CTC TTC TC-3′ (forward) and 5′- GTC GTG GTC AGT CAA CAT GG -3′ (reverse); 18 S ribosomal RNA (18S), 5′-CTG AGA AAC GGC TAC CAC ATC-3′ (forward) and 5′-CGC TCC CAA GAT CCA ACT AC-3′ (reverse). Data were analyzed with the Light Cycler 3.5 software. mRNA expression was standardized to 18S ribosomal RNA levels mRNA levels, as indicated in legends.

### Preparation of Tissues Protein Extracts and Western Blot Analysis

Following mouse tissues were collected: *M. oblongata*, spinal cord, the *gastrocnemius* muscle and the *L. dorsi* (broadest dorsal) muscle, liver and kidney. Total extracts, cytosolic fractions and crude membrane extracts were prepared as described previously (Canonne-Hergaux et al., 1999; Starzyński et al., 2009). Protein concentration was determined spectrophotometrically using the Bio-Rad protein assay. SOD1 was detected in total protein extracts obtained from tissues using rabbit polyclonal anti-superoxide dismutase 1 antibody (Abcam; ab16831). H- (H-Ft) and L-ferritin (L-Ft) subunit expression was analyzed using cytosolic extracts and rabbit antisera raised against, respectively, the recombinant mouse ferritin H and L subunits (kindly provided by Dr. Paolo Santambrogio, San Raffaele Scientific Institute, Milano, Italy). L-Ft was also analyzed using purified rabbit mouse liver ferritin antiserum (kindly provided by Dr. J. Brock, Glasgow University, Glasgow, UK) (Bouton et al., 2002). Fpn, Cp and HO1 levels in crude membrane extracts were detected using, respectively, a rabbit polyclonal antibody raised against mouse Fpn (MTP11-A, Alpha Diagnostics), a rabbit polyclonal antibody raised against human Cp (DAKO) and a rabbit polyclonal antibody raised against rat HO1 (ADI-OSA-150-F, Enzo Life Sciences). The loading controls – β-actin and tubulin in protein extracts were detected using goat polyclonal and mouse monoclonal antibodies, respectively (both from Santa Cruz Biotechnology). Peroxidase-conjugated anti-rabbit, anti-chicken, anti-mouse, or anti-goat secondary antibodies (Santa Cruz Biotechnology) were used. Immunoreactive bands were detected using the Western Bright ECL Western blotting detection kit (Advansta). Tissue extracts from *Hmox1^-/-^* or *Irp1^-/-^* mice or recombinant L- and H-Ft proteins (kindly provided by Dr. Paolo Santambrogio) were used as controls. Western blot analyses were performed using extracts prepared separately for each mouse (six experimental groups: two age groups and three genotypes for each age; three mice in each group; total 18 extracts per tissue).

### Statistical Analysis

All experiments were performed in biological triplicates, and error bars indicate standard deviation. Statistica 10 software was used for all of the statistical analyses. The assays were performed by two way ANOVA and means comparisons were adjusted by Tukey (*P* < 0.05 and *P* < 0.01).

## Results

### Increased SOD1 Expression and Activity in Tissues of Transgenic *SOD1* and *SOD1^G93A^* Mice

**FIGURE 1:**
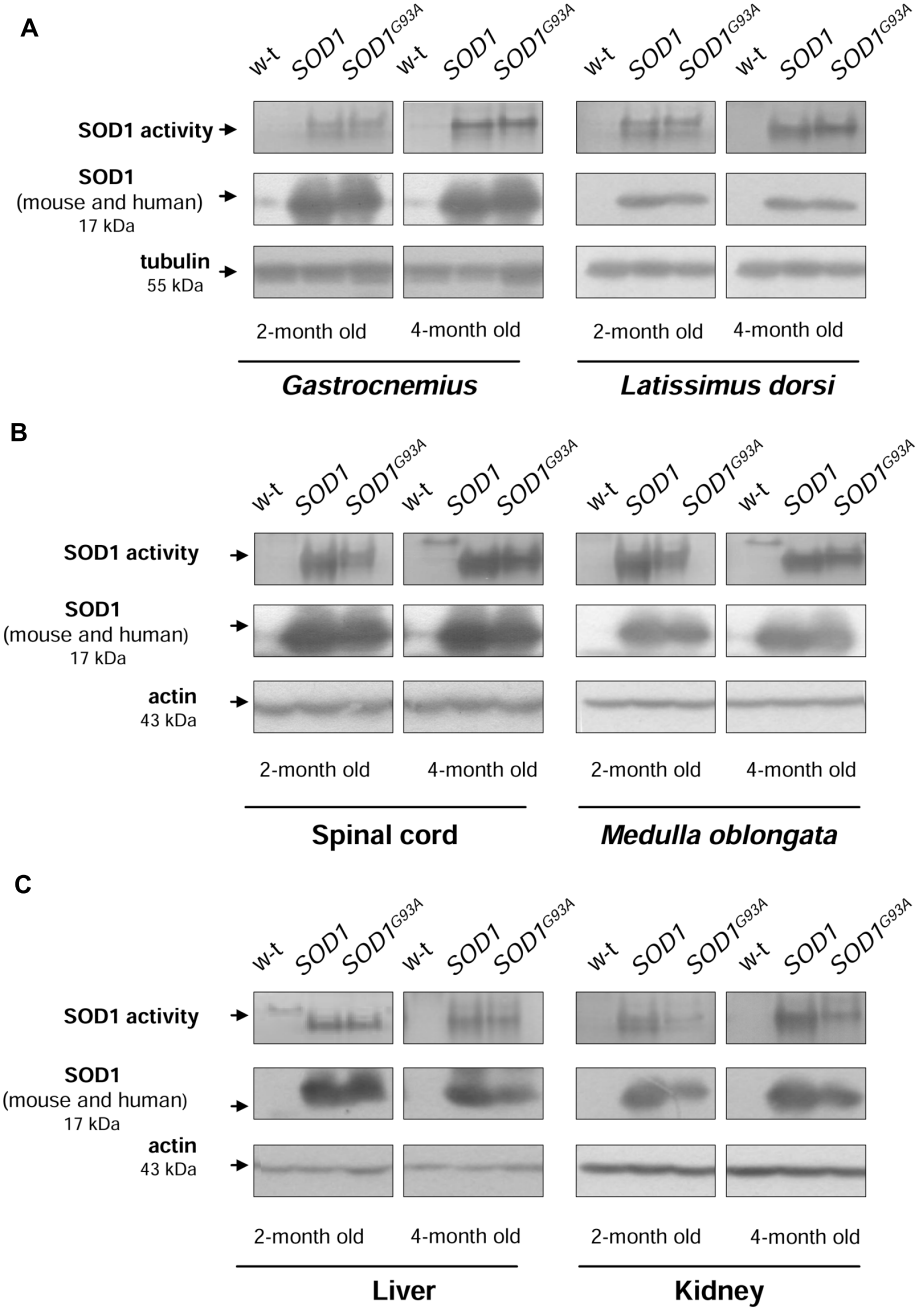
**CuZn-SOD1 (SOD1) activity and expression in tissues of experimental mice.** In-gel activity staining for SOD1 activity in total protein extracts obtained from skeletal muscles **(A)**, neuronal tissues **(B)**, liver, and kidney **(C)** (upper). Results of Western blot analysis of SOD1 in the same protein extracts are shown in (middle). Blots were also re-probed with monoclonal anti-tubulin or anti-β-actin antibodies from mouse as a loading control (lower). Data are representative for three independent Western blot analyses performed using extracts from 3 mice of each experimental group. The intensity of the SOD1 bands (relative to the intensity of tubulin or actin bands) was quantified with a Molecular Imager using Quantity One software (Bio-Rad) and is shown in arbitrary units to present protein level (Supplementary Table). Results are expressed as mean ± SD for three mice in each experimental group. Significant differences (*P* < 0.01) in SOD1 protein levels were found between transgenic (*SOD1* and *SOD1^G93A^*) and wild-type mice in a given tissue and age group.

Before investigating iron metabolism genes in mice from 3 experimental groups, first we measured SOD1 activity and expression in tissues affected by ALS (neuronal tissues and skeletal muscles) and in the two tissues important for systemic iron metabolism (liver and kidney). In all analyzed tissues: skeletal muscles (**Figure 1A**), neuronal tissues (**Figure 1B**), liver (**Figure 1C**, left-hand), and kidney (**Figure 1C**, right-hand) from both transgenic *SOD1* and *SOD1^G93A^* mice, the superoxide-scavenging activity of SOD1 was markedly higher compared to age-matched 2- and 4-month-old wild-type animals. We found that this increase was due to significantly (*P* < 0.01) elevated SOD1 protein level, detected by western immunoblotting (Supplementary Table). We found that in spinal cord, *M. oblongata* (**Figure 1B**), liver and kidney (**Figure 1C**) of the transgenic *SOD1* mice SOD1 protein was expressed at a higher level than in the corresponding tissues of the ALS mice although these differences did not reach statistical significance. Accordingly, the SOD1 superoxide-scavenging activity was higher in those tissues of mice overexpressing human wt *SOD1* gene.

### Pathological Changes and Iron Distribution in the *gastrocnemius* Muscle from Symptomatic ALS Mice

**FIGURE 2:**
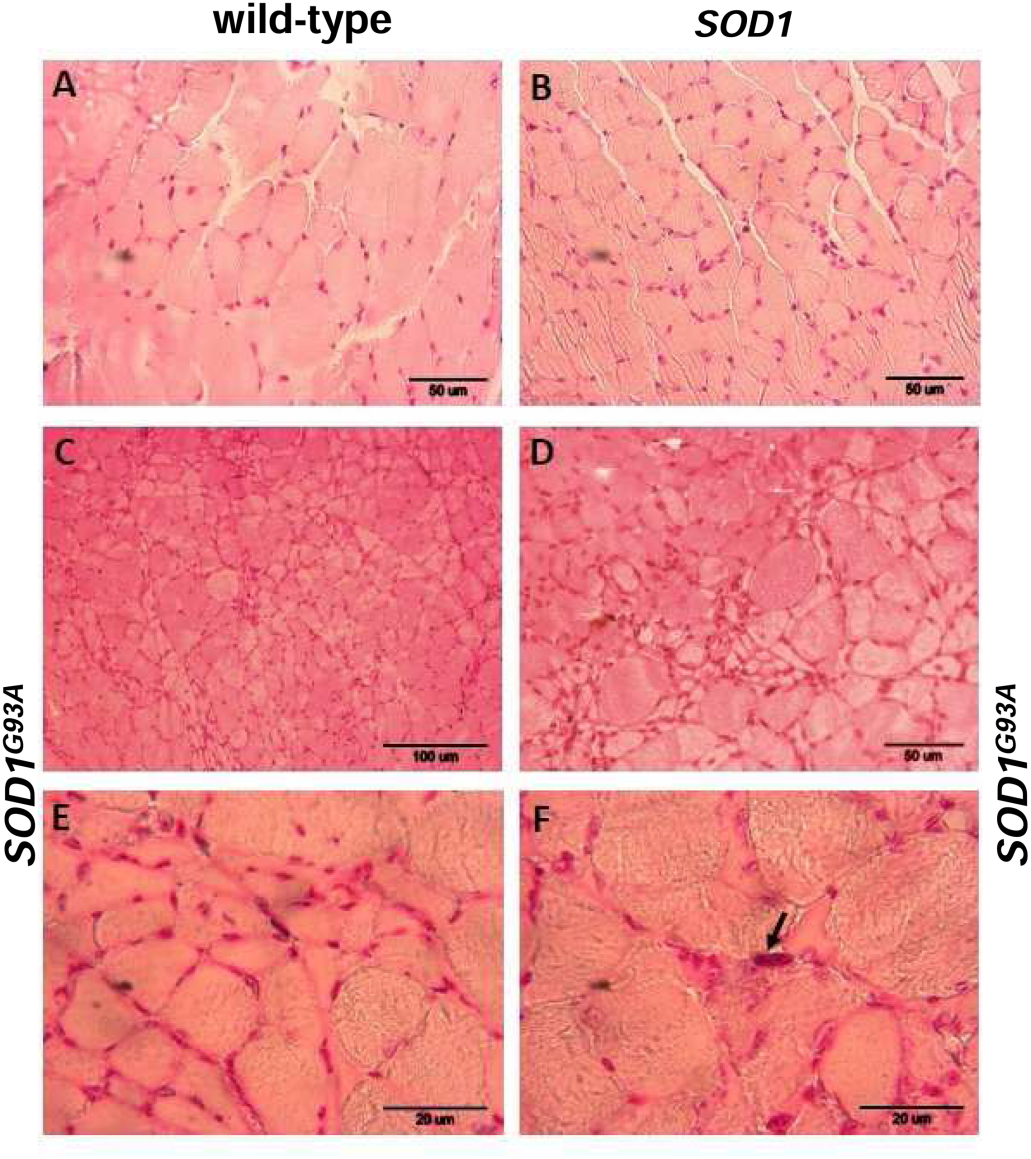
**Pathological changes in the *gastrocnemius* muscle of symptomatic ALS mice.** Haematoxylin and eosin-stained cross-sections of the gastrocnemius muscle from 4-month-old wild-type **(A)** and *SOD1* transgenic **(B)** mice show similar regular size of muscle fibers and normal architecture of the muscle; bar = 50 μm. Sections from aged-matched symptomatic *SOD1^G93A^* mice show pathological changes in muscle structure characteristic for ALS **(C–F)**. Larger regions of the muscle sections at lower magnification (bar = 100 μm) show characteristic grouped atrophy in the muscle of *SOD1^G93A^* mice **(C)**. Muscle tissue from symptomatic *SOD1^G93A^* mice shows the presence of mixed fiber types of different size. Group of small and atrophic muscle fibers and presence of hypertrophied fibers in the *gastrocnemius* muscle from 4-month-old symptomatic mouse **(D)**; bar = 50 μm. Group of the small and angular fibers at a higher magnification (bar = 20 μm) **(E)**. Pyknotic nuclear clumb (arrow) present in the muscle of symptomatic *SOD1^G93A^* mice **(F)**; bar = 20 μm.

**FIGURE 3:**
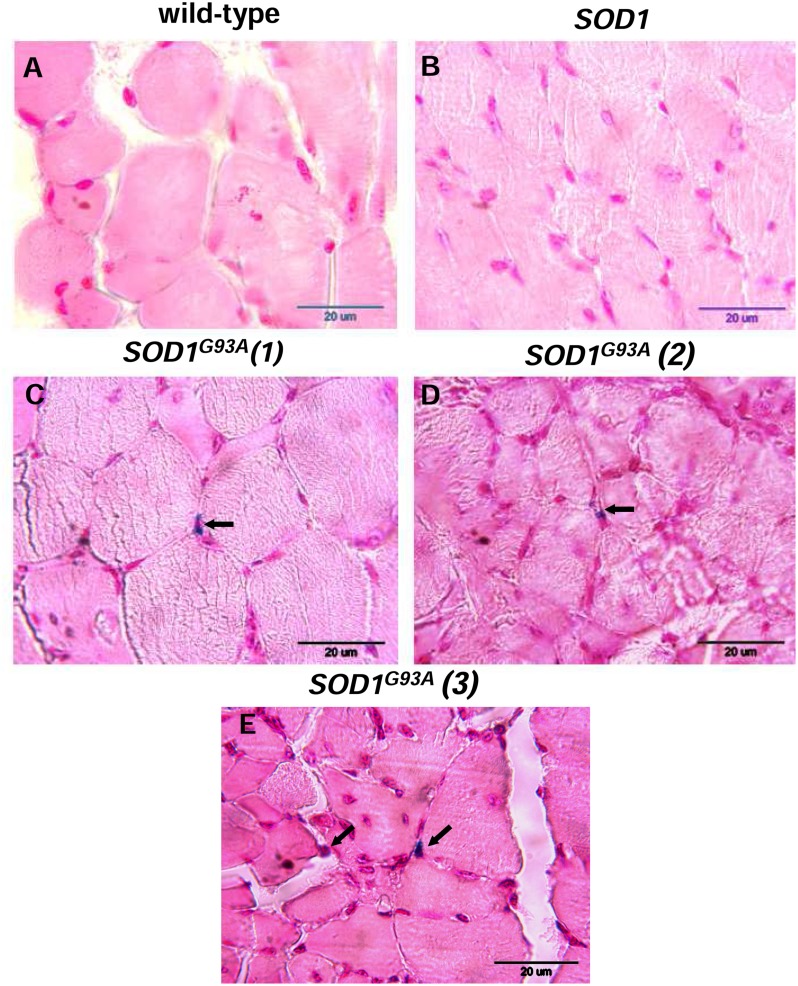
**Histological examination of iron loading in the *gastrocnemius* muscle of 4-month-old experimental mice.** Non-heme iron deposits were detected by staining with Perls Prussian Blue in the *gastrocnemius* muscle of wild-type **(A)** and *SOD1* mice **(B)**. Blue staining (indicated by arrows) was present exclusively in the muscle section of symptomatic *SOD1^G93A^* mice. Staining of muscle sections from three *SOD1^G93A^* mice are shown **(C,D,E)**.

Skeletal muscle atrophy observed in SOD1 models of ALS is usually considered as a consequence of motor neuron loss, although there is some evidence that it is a direct target of SOD1-mediated pathology (Dobrowolny et al., 2008). We analyzed myopathology of the gastrocnemius muscle upon staining with eosin/hematoxylin. We did not find any abnormalities in this muscle in 2-month-old mice – neither in the wild-type strain, nor in the transgenic ones – *SOD1* and *SOD1^G93A^* (data not shown). Similarly, no abnormalities were detected in 4-month-old wild-type and transgenic *SOD1* animals. In contrast, the *gastrocnemius* muscle from symptomatic transgenic *SOD1^G93A^* mice showed changes in the muscle architecture characteristic for ALS (**Figure 2F**). In particular, fibers had similar size (**Figures 2A,B**) in the muscle of 4-month-old control mice (wild-type and *SOD1*). In contrast, on the muscle section of the 4-month-old *SOD1^G93A^* mice we observed grouped angular atrophic muscle fibers (**Figure 2F**) and among small and degenerative muscle fibers we identified hypertrophic ones (**Figures 2C–E**). Few characteristic pyknotic nuclear clumbs were observed mainly in the group of degenerated fibers (**Figure 2F**). Microscopic analysis of gastrocnemius muscle sections from 4-month old wild-type and transgenic *SOD1* mice stained for non-heme iron with Perls’ Prussian blue showed no evidence of non-heme iron accumulation (**Figures 3A,B**). In contarst, non-heme iron accumulation was detected in the *gastrocnemius* muscles derived from 4-month-old *SOD1^G93A^* symptomatic mutants (**Figures 3C–E**). Deposits of iron had a form of irregular cytoplasmic inclusions, localized in some (not all) muscle fibers close the cell nuclei.

### IRP1 Protein Levels are Unaffected in *SOD1* and *SOD1^G93A^* Mice

**FIGURE 4:**
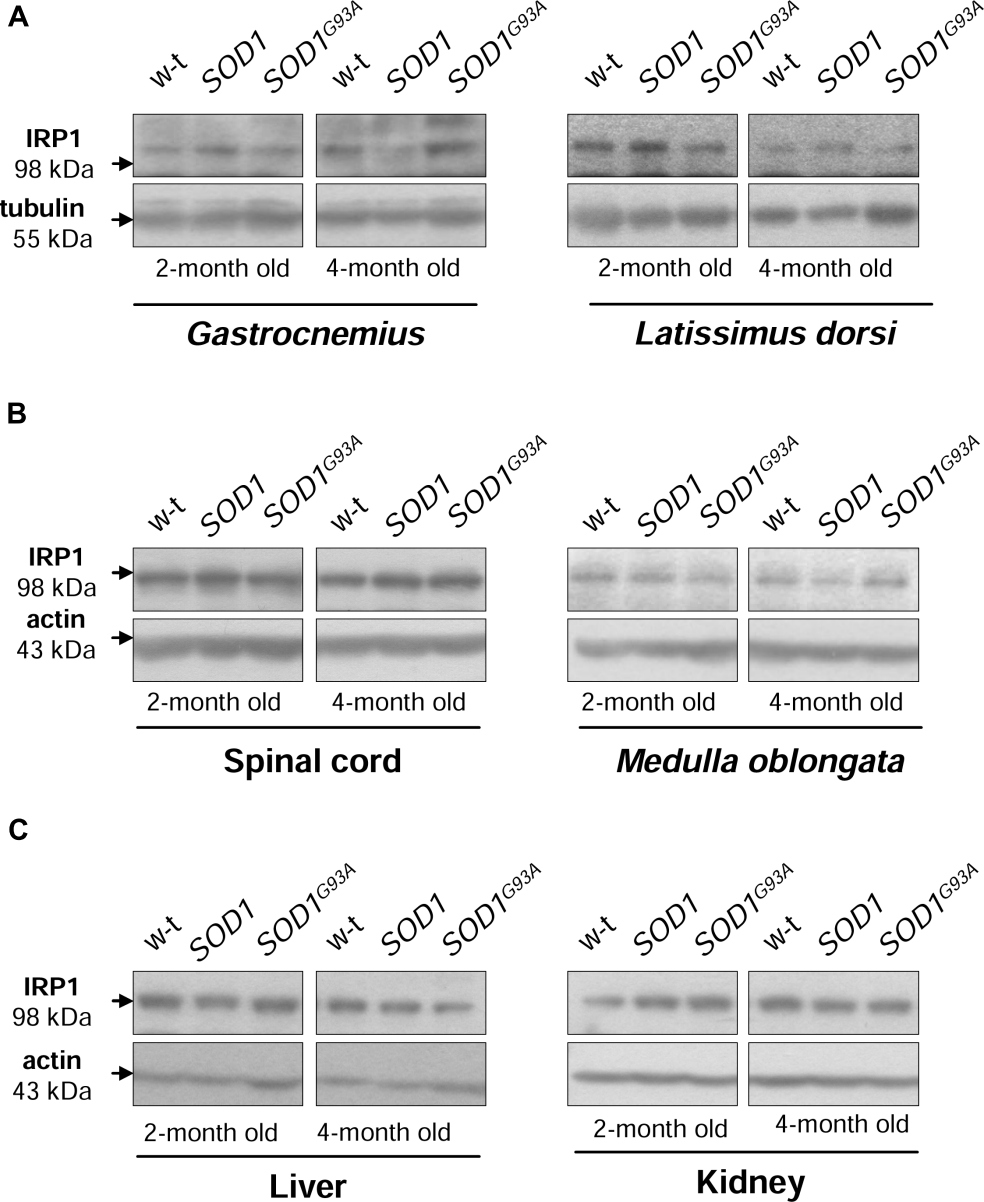
**Wild-type, *SOD1* and *SOD1^G93A^* mice show similar IRP1 protein levels in all examined tissues.** IRP1 levels were assessed in total skeletal muscle **(A)**, neuronal tissues **(B)**, hepatic and renal **(C)** extracts by Western blotting as described under “Material and Methods”. Blots were also re-probed with monoclonal anti-tubulin or anti-β-actin antibodies from mouse as a loading control. Data are representative for three independent Western blot analyses performed using extracts from 3 mice of each experimental group. The intensity of the IRP1 bands (relative to the intensity of tubulin or actin bands) was quantified with a Molecular Imager using Quantity One software (Bio-Rad) and is shown in arbitrary units to present protein level (Supplementary Table).

Iron regulatory protein 1 (IRP1) is a cytoplasmic bifunctional protein showing either aconitase or trans-regulatory activity involved in the mechanisms that control iron metabolism in mammalian cells. IRP1 lacking its iron-sulfur cluster acts as a trans-regulatory element that modulates the expression of iron-related proteins such as ferritin subunits, TfR1 and Fpn at a post-transcriptional level by binding to specific iron regulatory elements (IREs) on their mRNAs (Wilkinson and Pantopoulos, 2014). We have previously shown that in *SOD1* knock-out mice the expression of IRP1 is strongly reduced (Starzyński et al., 2005). On the other hand, the effect of increased SOD1 activity on IRP1 protein level has been investigated in cells overexpressing wild-type *SOD1* gene as well as in transgenic *SOD1^G93A^* and *SOD1^G37R^* ALS cellular (Danzeisen et al., 2006) and mouse (Massignan et al., 2007; Jeong et al., 2009) models, but discrepant data on IRP1 regulation have been reported. As intracellular protein IRP1 level is important for modifications of its IRE-binding affinity (Lipiński et al., 2000; Starzyński et al., 2006) we compared its expression at the protein level in cytosolic extracts obtained from tissues of wild-type, *SOD1* and *SOD1^G93A^* mice. Our Western blot analysis shows permanently high stability of IRP1 protein in all analyzed tissue tissues obtained from both asymptomatic and symptomatic *SOD1^G93A^* mice, in age-matched mice overexpressing wild-type *SOD1* gene and in wild-type controls (**Figure 4**). Our results militate against the possibility that in our model of ALS changes in IRP1 expression may account for its IRE-binding activity and in consequence for the regulation of its target mRNAs.

### Increased H-Ft Protein Levels in *SOD1* and *SOD1^G93A^* Mice

**FIGURE 5:**
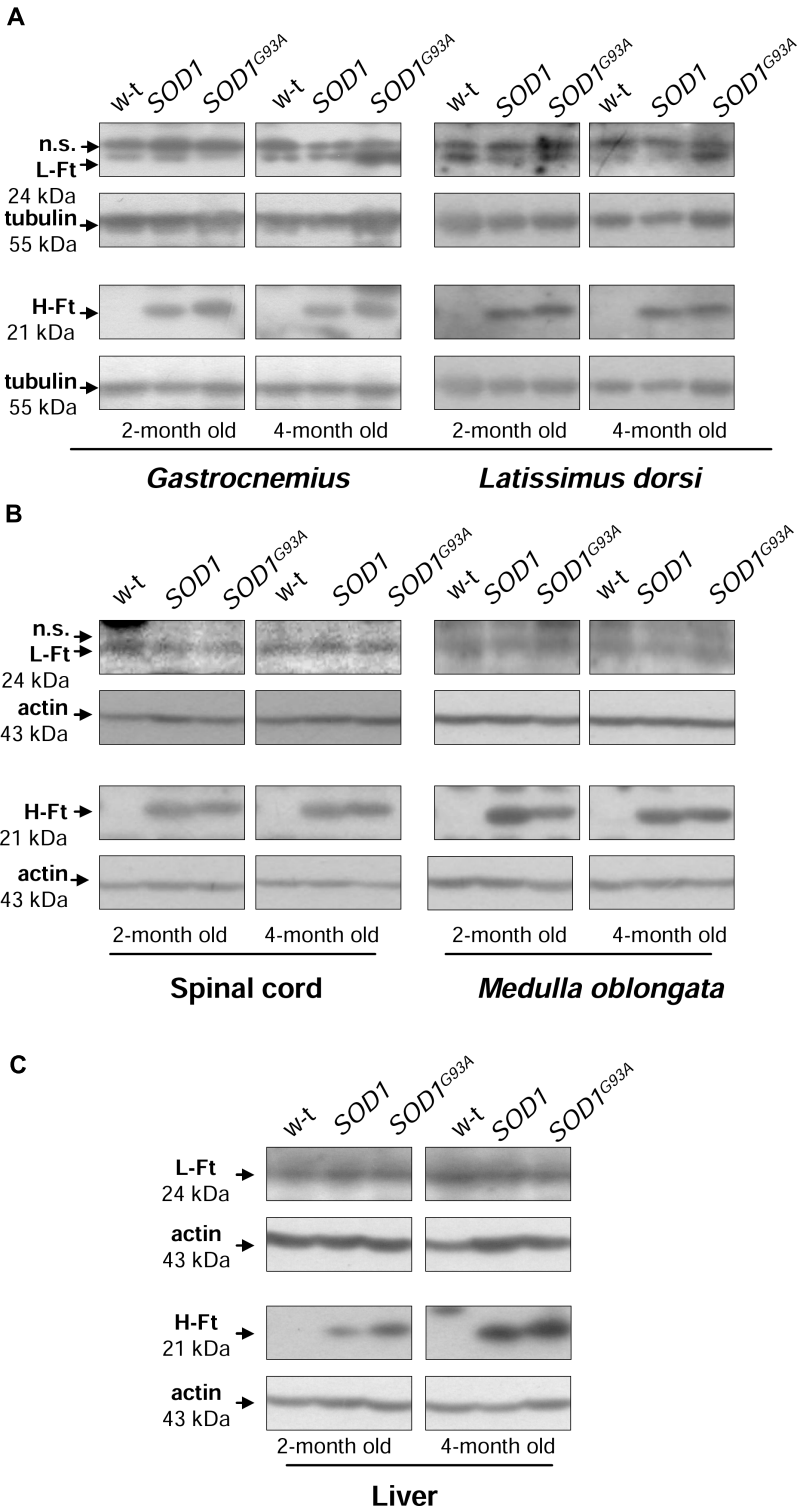
**Analysis of ferritin protein levels in tissues of experimental mice.** Western blot analysis of L-Ft and H-Ft protein levels in skeletal muscle **(A)**, neuronal tissues **(B),** and liver **(C)** cytosolic extracts. Tubulin and actin were used as a loading controls, as indicated. n.s. – non-specific band detected with purified rabbit mouse liver ferritin antiserum. Data are representative for three independent Western blot analyses performed using extracts from 3 mice of each experimental group. The intensity of the H- and L-Ft bands (relative to the intensity of tubulin or actin bands) was quantified with a molecular Imager using Quantity One software (Bio-Rad) and is shown in arbitrary units to present proteins level (Supplementary Table). Statistically significant differences (*P* < 0.01) in H-Ft protein levels were found between transgenic (*SOD1* and *SOD1^G93A^*) and wild-type mice in a given tissue and age group.

Ferritin level within the cell is a hallmark of intracellular iron accumulation (Harrison and Arosio, 1996). Among two types of subunits forming a hollow shell of the protein, H-ferritin possessing ferroxidase activity has been also shown to be regulated by oxidants and to prevent pro-oxidant labile iron from exacerbating oxidative stress (Orino et al., 2001). Bearing this in mind and referring to the concept of the involvement of oxidative stress in ALS pathology, we aimed to check whether changes in ferritin expression in tissues are specific for *SOD1^G93A^* mouse model of ALS. We determined both H- and L-ferritin expression at the protein level (**Figures 5A–C**). We noticed a substantial (from ∼3- to 25-fold depending on tissue, Supplementary Table) upregulation of H-ferritin protein in all analyzed tissues in both *SOD1* and *SOD1^G93A^* mice. This increase has been already observed in 2-month-old mice, strongly suggesting that it is rather caused by the enhanced SOD1 activity and is not directly linked strictly to the ALS pathology. In all tissues L-ferritin protein levels remained largely unaffected (**Figures 5A–C**).

### Similar Expression Patterns of Iron Importer and Exporter Genes in *SOD1*, *SOD1^G93A^* and Wild-type Mice

**FIGURE 6:**
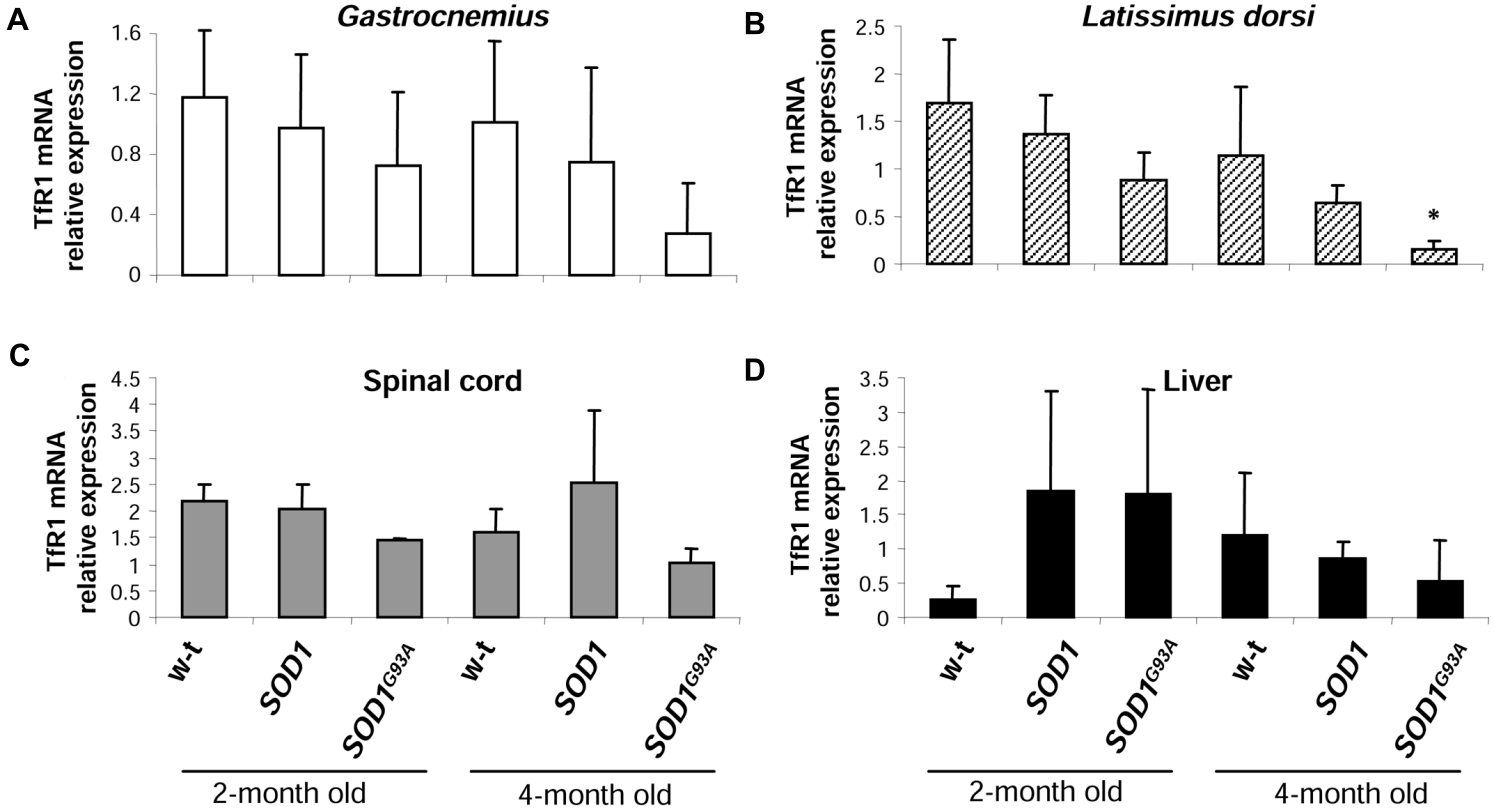
**Analysis of TfR1 mRNA abundance in tissues of experimental mice.** TfR1 mRNA abundance in skeletal muscles **(A,B)**, spinal cord **(C),** and liver **(D)** was measured by real-time RT-PCR as described under “Materials and methods.” The histograms display TfR1 mRNA levels after normalization to 18S ribosomal RNA levels. Each column represents the mean ± SD of three biological amplification reactions. RNA from each mouse was extracted separately using TRIZOL reagent, reverse-transcribed and amplified as described in M&M; therefore, 18 cDNA samples (biological triplicates for each condition) were finally used in the analysis of gene expression in each tissue. ^∗^, significant difference versus wild-type in the group of symptomatic, 4-month-old mice (*P* < 0.05).

**FIGURE 7:**
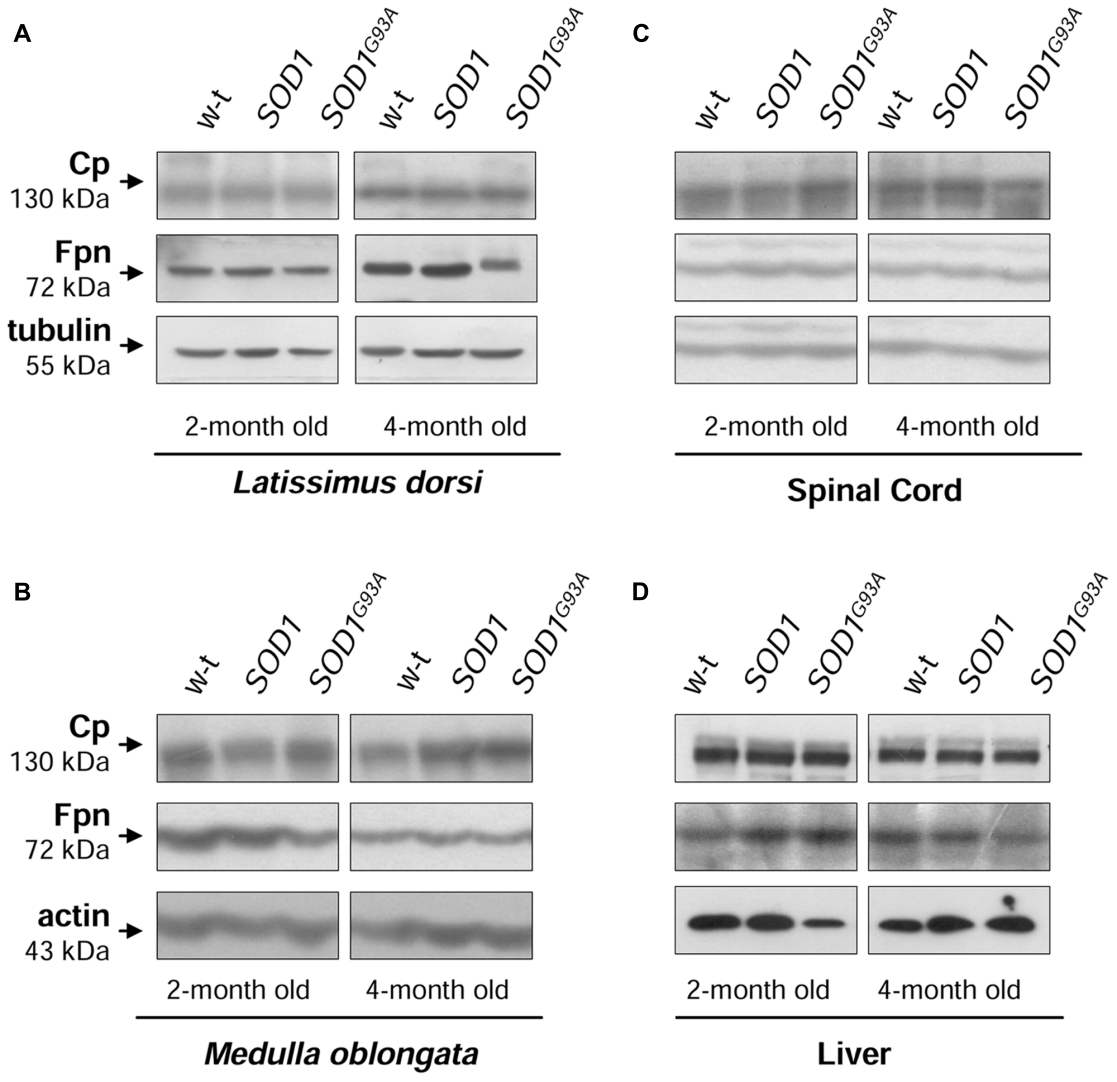
**Analysis of Cp and Fpn protein levels in tissues of experimental mice.** Western blot analysis of Cp and Fpn protein levels in the *Latissimus dorsi* muscle **(A)**, neuronal tissues **(B,C)**, and liver **(D)** membrane extracts Tubulin and actin were used as loading controls. Data are representative for three independent Western blot analyses performed using extracts from three mice of each experimental group). The intensity of the IRP1 bands (relative to the intensity of tubulin or actin bands) was quantified with a Molecular Imager using Quantity One software (Bio-Rad) and is shown in arbitrary units to present proteins levels (Supplementary Table).

The cellular iron status is largely influenced by the balance between iron influx to the cell and its export from the cell to the extracellular environment. Therefore in selected tissues (such as muscles, spinal cord, and liver) of mice from three experimental groups we determined the expression of the main iron importer gene, TfR1. Of importance, TfR1 is predominantly regulated at the level of its transcript stability *via* the post-transcriptional system IRP/IRE (Wilkinson and Pantopoulos, 2014). As show in **Figures 6A–D** the TfR1 mRNA levels measured by real-time quantitative RT-PCR show tendency to decrease in all analyzed tissues of *SOD1^G93A^* mice compared with wild-type and *SOD1* mice. However, the only statistically significant decrease in TfR1 mRNA level was found in the case of *L. dorsi* muscle of *SOD1^G93A^* symptomatic mice (**Figure 6B**). As regards proteins cooperating in iron export – Fpn and Cp, their levels in all analyzed tissues of *SOD1^G93A^* and *SOD1* mice assessed by Western blotting are the same as those found in control animals (wild-type and *SOD1*) in the two analyzed periods (**Figures 7A–D**).

### Heme Oxygenase 1 (HO1) mRNA and Protein Levels are Specifically Increased in Muscles and Spinal Cord of Symptomatic *SOD^G93A^* Mice

**FIGURE 8:**
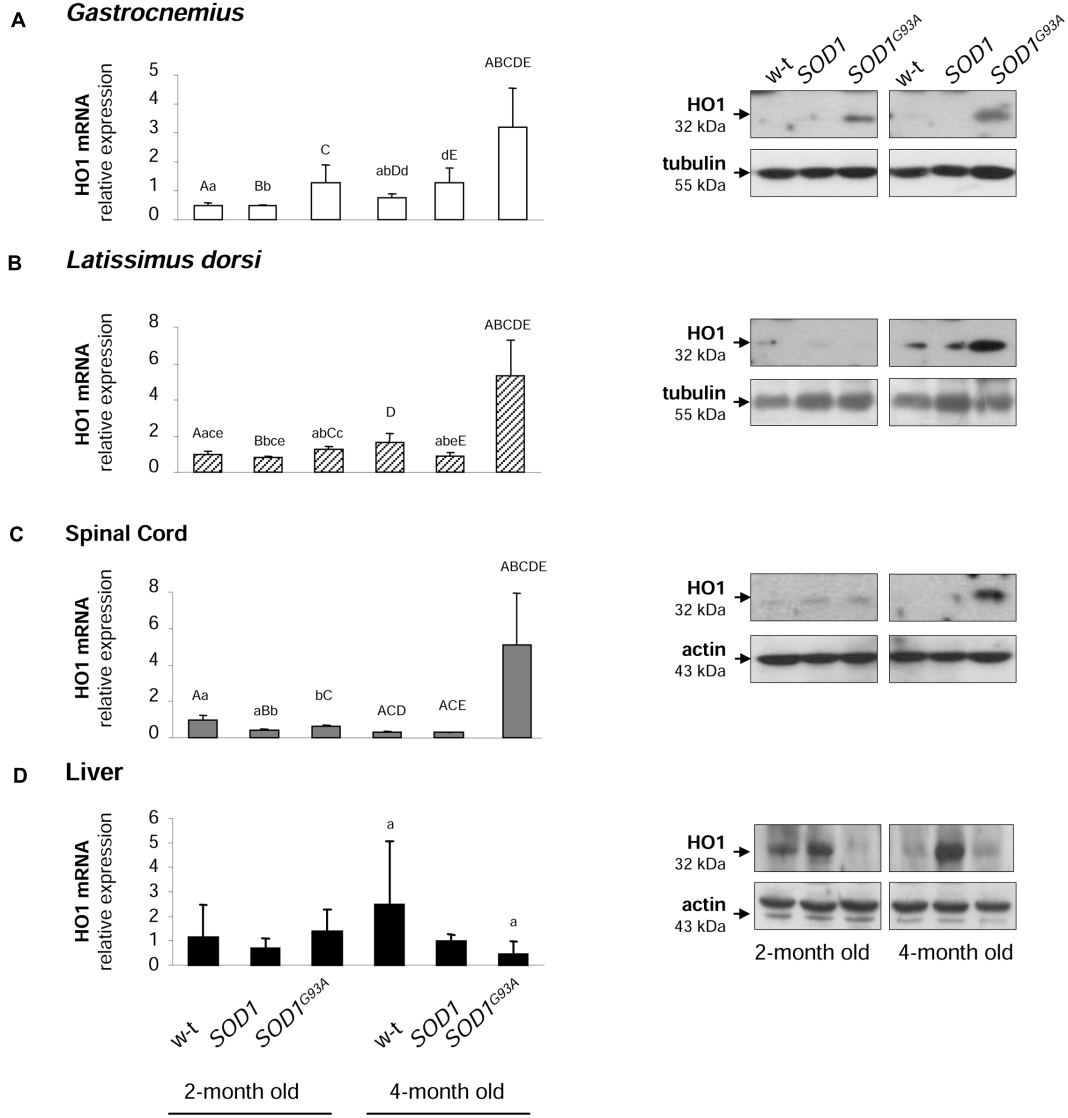
**Analysis of HO1 mRNA abundance and protein levels in tissues of experimental mice.** HO1 transcript abundance was measured in skeletal muscle **(A,B)**, neuronal tissues **(C)**, and liver **(D)** (left-hand) by real-time RT-PCR as described in M&M. The histograms display HO1 mRNA levels after normalization to 18S ribosomal RNA levels. Each column represents the mean ± SD of three biological amplification reactions. RNA from each mouse was extracted separately using TRIZOL reagent, reverse-transcribed and amplified as described in M&M; therefore, 18 cDNA samples (biological triplicates for each condition) were finally used in the analysis of gene expression in each tissue. The same capital and small letter indicates groups between which statistically significant differences were found at *P* < 0.01 and *P* < 0.05, respectively. Western blot analysis of HO1 protein levels was performed in membrane extracts obtained from the above-mentioned tissues (right-hand). Data are representative for three independent Western blot analyses performed using extracts from three mice of each experimental group. The intensity of the HO1 bands (relative to the intensity of tubulin or actin bands) was quantified with a molecular Imager using Quantity One software (Bio-Rad) and is shown in arbitrary units to present protein level. Results are expressed as mean ± SD for three mice in each experimental group.

Activation of HO1, a heme-degrading, inducible enzyme responsive to a wide range of cellular stimuli, is considered to convey adaptive responses to various stress conditions including oxidative stress (Grochot-Przeczek et al., 2012). Surprisingly, HO1 expression has been intermittently investigated in ALS pathology (Dwyer et al., 1998). Here we show that *Hmox1* gene is specifically induced at both mRNA and protein levels in muscles (**Figures 8A,B**) and spinal cord (**Figure 8C**) of symptomatic *SOD^G93A^* mice. In the *gastrocnemius* muscle the up-regulation of *Hmox1* gene was already observed in asymptomatic mice (**Figure 8A**). Importantly, in the liver of *SOD1^G93A^* mice, tissue not affected by ALS pathology, we did not observe any HO1 up-regulation, neither at the mRNA nor at the protein levels (**Figure 8D**).

## Discussion

Among antioxidant enzymes the family of ubiquitous SODs deserves special attention because the reaction catalyzed by SODs converts one ROS (O_2_^⋅-^) to another (H_2_O_2_), and thus these enzymes play a subtle role in the regulation of ROS balance (Zelko et al., 2002). Accordingly, both deficiency and overexpression of Cu,Zn-SOD (SOD1), mostly cytosolic enzyme, induce oxidative stress in a wide range of organisms (Reaume et al., 1996; Lee et al., 2001; Missirlis et al., 2003; Starzyński et al., 2009). On the other hand, it is tempting to propose that enhanced SOD1 activity may lead to the excessive H_2_O_2_ formation, and subsequent iron-related exacerbation of oxidative stress through the redox reaction of H_2_O_2_ with ferrous iron but also through the H_2_O_2_-dependent disturbance of iron homeostasis regulatory mechanisms (Orino et al., 2001).

Identification of mutations in *SOD1* gene as causative ones in familial amyotrophic lateral sclerosis (fALS) (Rosen et al., 1993) was immediately followed by the generation of transgenic mice constitutively overexpressing mutated human SOD1 (Gurney et al., 1994). Nowadays these animals are widely used in ALS research (Turner and Talbot, 2008). However, investigation of some pathogenic aspects of this disease in rodent models of mutant SOD1-mediated fALS requires special consideration of increased SOD1 activity, which does not occur in human pathology. It seems that this remark is of particular importance when studying a potential role of iron in the pathogenesis of ALS. Misregulated iron homeostasis that promotes excessive oxidative stress in the motor neurons has been postulated to contribute to the disease pathogenesis (Carri et al., 2003). This proposal is now strengthened by several lines of evidence coming from studies on ALS patients (Kasarskis et al., 1995; Mitchell et al., 2010; Kwan et al., 2012; Ignjatović, 2013), animal ALS models (Jeong et al., 2009; Wang et al., 2011; Lee et al., 2015) and cellular ALS models (Danzeisen et al., 2006; Hadzhieva et al., 2013). Transgenic *SOD1^G93A^* (Wang et al., 2011; Lee et al., 2015) and *SOD1^G37R^* (Jeong et al., 2009) mice, abundantly expressing active mutant SOD1 and showing fast and slow progression of the disease, respectively, are the most intensely studied *in vivo* models for investigating the contribution of iron to the pathogenesis of ALS. However, in these studies the results obtained from mice overexpressing mutated *SOD1* gene were not compared with those from transgenic mice overexpressing human wild-type *SOD1*.

In our study, we adopted an experimental approach aimed at comparing changes in iron metabolism in 2-month-old and 4-month-old age-matched mice carrying mutated *SOD1^G93A^* and non-mutated human *SOD1* genes, as well as in control non-transgenic mice. We show that in all analyzed tissues the expression and SOD1 activity was similar in both *SOD1^G93A^* and *SOD1* mice and highly raised in both of the transgenic strains in comparison to the wild-type animals. Our results stay in accordance with the prediction of a high copy number of the transgene in *SOD1^G93A^* mice (Gurney et al., 1994). Considering the evidence that skeletal muscle weakness and atrophy, followed by paralysis is a final major cause of disability and death in ALS (Pratt et al., 2012; Pansarasa et al., 2014), we first wanted to determine histological hallmarks of myopathology in mice from three experimental groups. Importantly, we found pathological changes in the *gastrocnemius* muscle morphology only in symptomatic *SOD1^G93A^* mice, which indicates that the enhanced activity of SOD1 in those animals is not sufficient for the induction of skeletal muscle pathology, and which confirms progressive character of the disease. Our findings corroborate results of a comprehensive examination of various aspects of muscle pathology from animal SOD1 models of ALS (Pansarasa et al., 2014) and from clinically confirmed ALS patients (Léger et al., 2006). The impairment of iron metabolism that leads to excessive accumulation of this metal in tissues altered in ALS has been mainly demonstrated in relation to neuronal system (Kasarskis et al., 1995; Kwan et al., 2012; Ignjatović, 2013). Here, we provide histological evidence that among mice from 3 experimental groups analyzed at two stages of the disease, deposits of iron were strictly specific for skeletal muscles of symptomatic *SOD1^G93A^* animals. Accordingly, progressive increase in muscle iron content has been shown in *SOD1^G93A^* rats from the disease onset up to the end-stage of ALS (Halon et al., 2014).

Enhanced ferritin level reflects iron accumulation but may be also considered as an element of the adaptive response to oxidative stress that limits the bioavailability of the free iron, which participates in the generation of ⋅OH. Our analysis of H- and L-ferritin expression clearly shows that only H-Ft is upregulated at the protein level in *SOD1^G93A^* mice, not only in tissues primarily affected by ALS (muscles, *M. oblongata*, and spinal cord) but also in the liver, a tissue, which is not directly related to the ALS pathology. Unexpectedly, similar increase in H ferritin chains expression was also observed in all analyzed tissues of mice that overexpress a wild-type human *SOD1* gene. This uniform ferritin upregulation in *SOD1^G93A^* and *SOD1* mice strongly suggests that it results from oxidative stress induced by increased SOD1 activity. Although increased ferritin expression has been reported in skeletal muscles of *SOD1^G93A^* rats (Halon et al., 2010, 2014), in spinal cord of *SOD1^G93A^* mice (Massignan et al., 2007) and microglia of *SOD1^G37R^* mice (Jeong et al., 2009), the question of the specificity of this upregulation associated with ALS pathology remains uncertain as in all those studies only wild-type animals were used as controls. Ferritin is commonly considered as a hallmark of cellular iron status, and an increase in ferritin mirrors iron accumulation. Our observation of both iron deposits and high ferritin levels in the *gastrocnemius* muscle of symptomatic *SOD1^G93A^* mice seems to confirm this rule. On the other hand, as shown in this study, high level of cytosolic ferritin in the *gastrocnemius* muscle of *SOD1* mice is not associated with detectable iron deposits and, as shown by others, iron accumulation in neurons of *SOD1^G37R^* mice is not correlated with any increase in intracellular ferritin content (Jeong et al., 2009). All these data strongly suggest that ferritin may not be a valuable indicator of intracellular iron status in SOD1 ALS models. Regulation of ferritin subunits is mediated by various factors at various regulatory levels such as transcription (Torti and Torti, 2002), translation (Wilkinson and Pantopoulos, 2014) and protein stability (De Domenico et al., 2006). In addition, both ferritin subunits are up-regulated by iron *via* iron regulatory proteins (IRP1 and IRP2) (Wilkinson and Pantopoulos, 2014). We have previously shown that IRP1 protein level is strongly (by 80%) down-regulated in SOD1 knockout mice (Starzyński et al., 2005). Here, we demonstrate that under the opposite conditions of a marked increase in SOD1 activity – in *SOD1^G93A^* and *SOD1* mice, IRP1 level is unchanged. Accordingly, analysis of IRP1 in the spinal cord of *SOD1^G93A^* mice (Jeong et al., 2009) and in U373 MG glial cells overexpressing *SOD1^G93A^* and wild-type *SOD1* (Danzeisen et al., 2006) show no changes in IRP1 protein expression (based on the EMSA assay with 2% ME). In contrast to these findings, proteomic analysis of the spinal cord of presymptomatic ALS *SOD1^G93A^* mice demonstrated nearly threefold reduction in IRP1 protein level when compared to wild-type mice. However, this decrease has not been validated by direct evaluation of IRP1 level by Western blot analysis (Massignan et al., 2007). Interestingly, the two aforementioned studies (Danzeisen et al., 2006; Jeong et al., 2009) show that increased activity of SOD1 results in the activation of IRP1, i.e., in inducing its ability to bind to the IRE (iron responsive element) sequences contained in mRNAs encoding several iron-related proteins such as ferritin chains, TfR1 and Fpn (Wilkinson and Pantopoulos, 2014). It is tempting to propose that such regulation of IRP1 IRE-binding activity is mediated by H_2_O_2_ (Martins et al., 1995; Pantopoulos and Hentze, 1995) produced under conditions of increased SOD1 activity. Nevertheless, according to the principles of the IRP/IRE system, this increased binding of IRP1 to the IREs located in the 5′UTR of H-Ft, L-Ft and Fpn mRNAs, should inhibit their translation (Wilkinson and Pantopoulos, 2014). Therefore, considering that ferritin has been shown to be increased in animals and cells overexpressing *SOD1^G93A^*, multiple but yet unknown mechanisms may override the IRP1-mediated Ft inhibition in ALS.

TfR1 is a cell surface protein, providing iron bound to transferrin to the cells *via* endocytosis (Pantopoulos et al., 2012). Its expression is regulated by the IRP/IRE system in an opposite manner to Ft and Fpn. This means that activated IRP1 stabilizes TfR1 transcript by binding to several IRE motifs located in its 3′UTR (Wilkinson and Pantopoulos, 2014). The expression of TfR1 transcript in all analyzed ALS target tissues, such as skeletal muscles (*gastrocnemius* and *L. dorsi)* and spinal cord of *SOD1^G93A^* mice shows the tendency to be decreased when compared with *SOD1* and WT animals. Results of previous studies showing the TfR1 expression in SOD1 ALS animal and cellular models are not conclusive: cellular studies report the increase of TfR1 at both mRNA and protein levels (Danzeisen et al., 2006; Hadzhieva et al., 2013), but TfR1 protein expression in cervical cord of *SOD1^G37R^* mice has been shown unchanged (Jeong et al., 2009). Again, as in the case of Ft, the increase in TfR1 levels were reported in human glioblastoma astrocytoma cells overexpressing mutated and WT *SOD1* gene, which denotes no specificity of the regulation for the ALS pathology (Danzeisen et al., 2006).

An alternatively spliced, glycosylphosphatidylinositol (GPI) anchored form of Cp is a copper-dependent ferroxidase that oxidizes ferrous iron exported from the cells by Fpn, and thus enables binding of ferric iron to transferrin. The cooperation of this protein tandem is crucial for protecting the central nervous (CNS) system from toxic iron deposition. Accordingly, aceruloplasminemia in humans results in iron accumulation in the CNS and neurodegeneration (Hellman and Gitlin, 2002). Likewise, reduced Fpn expression was also associated with increased iron content in central nervous system cells under inflammatory conditions (Urrutia et al., 2013). Here, we show that neither the expression of GPI-Cp nor that of Fpn is altered in skeletal muscles and spinal cord of *SOD1^G93A^* mice. A recent study showed Fpn decrease in the *soleus* but not in the *tibialis* muscle of *SOD1^G93A^* rats (Halon et al., 2014). Authors argue that Fpn downregulation in the *soleus* muscle is due to the increased hepcidin, a peptide that binds Fpn and induces its degradation (Ganz and Nemeth, 2012). However, they do not explain why the systemic effect of hepcidin activity is restricted only to the one type of muscles. As regards Cp, to our knowledge, the expression of membrane anchored form was not studied in ALS, however, increased concentration of its soluble form (sCp, especially relative abundance of more basic sCp isoforms) has been reported in cerebrospinal fluid of ALS patients (Conti et al., 2008). Although serum Cp pattern has been proposed as disease feature, our results do not support the role of Cp in ALS pathogenesis.

The rationale for analyzing the expression of HO1 encoded by *Hmox1* gene in both *SOD1* and *SOD1^G93A^* mice is that this ubiquitous enzyme is induced by a great number of stress stimuli (including oxidative stress) and that is strictly connected with intracellular and systemic iron metabolism (Kovtunovych et al., 2010; Starzyński et al., 2013). HO1 mediates a broad range of beneficial effects by catalyzing the first and rate-limiting step in the heme degradation pathway, resulting in the formation of biologically active molecules such iron, carbon monoxide (CO) and biliverdin (Grochot-Przeczek et al., 2012). In consequence, increased HO1 expression has been considered as a cytoprotective, adaptive cellular response under conditions of oxidative stress and inflammation occurring *inter alia* in several neurodegenerative diseases (Schipper et al., 2009). Importantly, in this study we have shown that HO1 expression is increased in tissues of the transgenic *SOD1^G93A^* mice. Among few genes fundamental for cellular iron homeostasis and analyzed in this study, the upregulation of *Hmox1* gene seems to be strictly specific for skeletal muscles and neuronal tissues of symptomatic *SOD1^G93A^* mice. The only exception is *gastrocnemius* muscle, where HO1 protein level is increased also in asymptomatic mice overexpressing mutant SOD1. Of importance, this muscle, in contrast to the broadest dorsal muscle, is made up of mostly fast twitch (type II) muscle fibers, that are preferentially affected by ALS (Atkin et al., 2005). Therefore, it is tempting to speculate, that the oxidative stress precedes the onset of iron metabolism dysregulation in ALS. In contrast to our results, previous studies do not provide any evidence of increased HO1 expression in the spinal cord of *SOD1^G93A^* mice (Dwyer et al., 1998). The reason of this discrepancy is not known. Our analysis of *Hmox1* gene expression in *SOD1^G93A^* mice includes the evaluation of both HO1 mRNA and protein levels in various tissues and unambiguously shows a homogenous pattern of *Hmox1* gene induction. It has been postulated that cytoprotective effect of HO1 requires the co-induction of H-ferritin chain, which limits the pro-oxidant effect of the free iron released from the protoporphyrin IX ring of heme following HO1-mediated enzymatic reaction (Gozzelino and Soares, 2014). Coming back to the concept of the iron-mediated toxicity in ALS, it is tempting to propose that in *SOD1^G93A^* mice HO1 acts together with ferritin to limit the reactivity of heme-derived intracellular iron as it was originally demonstrated in endothelium under hemolytic conditions (Balla et al., 1992).

## Author Contributions

AG supported the design of the study, performed the research, and analyzed data. AS designed and performed the research, analyzed data, and contributed to the writing of the paper. RS contributed to the conception of the work, performed the research and analyzed data. AB performed the research and analyzed data. ML supported the design of the study, performed the research, revised the work for important intellectual content. RS performed the research, analyzed data, and drafted the work. PL designed the study and wrote the paper. All authors read and approved the final manuscript.

## Conflict of Interest Statement

The authors declare that the research was conducted in the absence of any commercial or financial relationships that could be construed as a potential conflict of interest.

## Abbreviations

ALS: amyotrophic lateral sclerosis
Cp: ceruloplasmin
Fpn: ferroportin
H-Ft: H feritin chain
L-Ft: L ferritin chain
HO1: heme oxygenase 1
IRP1: iron regulatory protein 1
SOD: superoxide dismutase
TfR1: transferrin receptor 1
