# Supplementary material for: Mice Overexpressing Both Non-Mutated Human SOD1 and Mutated SOD1G93A Genes: A Competent Experimental Model for Studying Iron Metabolism in Amyotrophic Lateral Sclerosis
Source: Front Mol Neurosci. 2016 Jan 6;8:82. doi: 10.3389/fnmol.2015.00082 (PMC4701970; doi:10.3389/fnmol.2015.00082)
Supplement: Supplementary file 1 [file Table_1.DOC]

|  |  | **presymptomatic (2 - month old)** | | | **symptomatic (4 - month old)** | | |
| --- | --- | --- | --- | --- | --- | --- | --- |
| **tissue** | **protein** | **w-t** | ***SOD1*** | ***SOD1^G93A^*** | **w-t** | ***SOD1*** | ***SOD1^G93A^*** |
| *Spinal cord* | SOD1 | 83.8±5.9 | 516.6±5.2** | 490.5±5.54** | 56.1±0.2 | 616.5±53.3** | 501.4±21.3**^#^ |
| *Medulla oblongata* | SOD1 | 4.1±0.56 | 211.8±29.5** | 182.1±43** | 42.7±8.1 | 250.3±41.6** | 224.7±103.2** |
| *Liver* | SOD1 | 11.5±5.58 | 412.7±104.7** | 334.3±68.1** | 141.8±10.8 | 489.1±111.3** | 280.9±22.2**^#^ |
| *Kidney* | SOD1 | 0.9±0.51 | 106.9±27.6** | 84.2±15.1**^#^ | 16.1±3.5 | 131.8±30.5** | 115.4±24.6** |
| *Gastrocnemius* | SOD1 | 35.2±10.5 | 276.3±6.1** | 242±6.9** | 51.3±0.9 | 257.5±18.4** | 275.3±16.6** |
| *Latissimus dorsi* | SOD1 | 0.2±0.03 | 80.1±11** | 81.7±20.2** | 19.1±7.1 | 81±20.3** | 105.2±29.1** |
| *Gastrocnemius* | HFT | 4.2±6 | 39.6±4.1** | 49±1.5**^#^ | 13.3±7.2 | 36.8±4.8* | 45.2±0.2*^#^ |
| *Latissimus dorsi* | HFT | 1.1±1.06 | 34.9±7.5** | 35.8±8.7** | 10.2±4.3 | 77.1±34.2* | 106.9±42.1*^#^ |
| *Spinal cord* | HFT | 6.89±5.51 | 172.3±42** | 158.3±27.8** | 6.54±7.6 | 157.1±17.8** | 171.2±35.7** |
| *Medulla oblongata* | HFT | 21.7±11.28 | 98.9±15.9* | 99.9±11.2* | 17.1±13.1 | 107.2±6.7** | 109.2±6.4** |
| *Liver* | HFT | 5.9±0.8 | 86.3±6.6** | 90.2±5.8** | 9.6±1.6 | 112.9±22.6** | 114.1±1.8** |
| *Gastrocnemius* | LFT | 100 ±8.4 | 102.3±6.7 | 100.4±5.7 | 107.9±7.3 | 95.4±9.1 | 119±2.7 |
| *Latissimus dorsi* | LFT | 61.9±24.4 | 55.6±18.5 | 69.4±21.1 | 24.3±11.6 | 22.5±7.6 | 47.3±22.3^#^ |
| *Spinal cord* | LFT | 18.6±8.5 | 13.1±6.1 | 12.6±4.9 | 13.4±9 | 12.3±8.4 | 12±6.6 |
| *Medulla oblongata* | LFT | 16.7±7.3 | 14.8±11.9 | 20.8±15.2 | 15.9±7.1 | 13.9±3.3 | 16.4±29.1 |
| *Liver* | LFT | 78.3±9.18 | 79.2±11.5 | 78.5±10.3 | 118.5±17.3 | 104.8±21 | 120.7±16.4 |
| *Gastrocnemius* | IRP1 | 69.1±10.7 | 92.3±11.8 | 64±4.1 | 108.1±8.3 | 79.1±6.5 | 107.9±18.8^#^ |
| *Latissimus dorsi* | IRP1 | 149±23 | 161.4±20.6 | 102.3±6.6^#^ | 98.5±7.6 | 118.9±9.7 | 75.5±13.1^#^ |
| *Spinal cord* | IRP1 | 134.8±27.2 | 130.7±19 | 105.2±11.4 | 100.1±2.4 | 117.2±2.3 | 119.7±3.4 |
| *Medulla oblongata* | IRP1 | 78.2±15.8 | 57.6±8.4 | 41.8±4.5* | 50.8±1.2 | 29.1±0.6* | 53±1.5^#^ |
| *Liver* | IRP1 | 139.9±14.6 | 105.6±3.4 | 131.3±20.7 | 111.3±8.1 | 95±8.9 | 66.6±3.3^#^ |
| *Kidney* | IRP1 | 77.6±8.1 | 123.4±4 | 132.4±20.9 | 142.5±10.4 | 124.6±11.6 | 122.4±6.1 |
| *Latissimus dorsi* | FPN | 138.8±6.4 | 127.4±5.6 | 140.1±9.1 | 146.1±11.2 | 155.2±5.4 | 111.3±8.6 |
| *Spinal cord* | FPN | 90.9±1.4 | 95.9±2.6 | 87.9±3.2 | 84.65±3.1 | 101.3±6.2 | 88.9±2.2 |
| *Medulla oblongata* | FPN | 92.7±5.5 | 95.6±4.2 | 69±7.1 | 62.7±2.1 | 58±1.4 | 55.2±3.1 |
| *Liver* | FPN | 103.8±3.1 | 117.7±8.2 | 189.1±20.3*^#^ | 140.3±11.1 | 109.4±10.5 | 105.2±8.2 |
| *Latissimus dorsi* | CP | 151.5±0.4 | 138.4±0.7 | 163.3±0.1 | 155.6±0.2 | 152.4±0.4 | 149.4±0.8 |
| *Spinal cord* | CP | 210±3.1 | 188.3±5 | 202.6±1.5 | 213.5±8.2 | 250.5±20.6 | 209.8±0.4^#^ |
| *Medulla oblongata* | CP | 99.9±0.5 | 95.1±0.2 | 98.5±0.1 | 87.1±0.1 | 97.2±0.1 | 106.6±3.6 |
| *Liver* | CP | 99.4±6.2 | 109.2±7.8 | 157.2±12.8*^#^ | 123±11.5 | 95.7±6.5 | 85.4±11.6 |
| *Gastrocnemius* | HO1 | 3.46±0.5 | 3.54±2.1 | 5.87±1.9*^#^ | 4±0.9 | 3.16±0.4 | 7.39±4.6*^#^ |
| *Latissimus dorsi* | HO1 | 2.95±1.7 | 0.65±0.6 | 0.1±0.05 | 4.45±0.8 | 6.51±4.4 | 13.99±4.2**^#^ |
| *Spinal cord* | HO1 | 0.4±0.02 | 1.07±0.6 | 0.83±0.41 | 0.3±0.18 | 1.62±1.4 | 8.88±2.2**^#^ |
| *Liver* | HO1 | 10.5±3.82 | 10.6±2.51 | 6.2±6.06*^#^ | 6.3±3.67 | 12.3±2.19* | 5.5±1.82^#^ |

**Supplementary Table.**

**Supplementary Table**. **Densitometric analysis of proteins detected by western immunoblotting.** The intensity of the proteins bands (relative to the intensity of tubulin or actin bands) was quantified with a Molecular Imager using Quantity One software (Bio-Rad) and is shown in arbitrary units to present proteins level. Results are expressed as mean ± S.D. for 3 mice in each experimental group. * (*P*<0.05) , ** (*P*<0.01) significant difference *versus* wild-type in the same age group. ^#^, significant difference versus *SOD1* in the group of 2- and 4-month old *SOD1^G93A^* mice *(P<0.05)*.
